# Activity and abundance of methane-oxidizing bacteria in secondary forest and manioc plantations of Amazonian Dark Earth and their adjacent soils

**DOI:** 10.3389/fmicb.2014.00550

**Published:** 2014-10-22

**Authors:** Amanda B. Lima, Aleksander W. Muniz, Marc G. Dumont

**Affiliations:** ^1^Department of Biogeochemistry, Max Planck Institute for Terrestrial MicrobiologyMarburg, Germany; ^2^Department of Soil Microbiology and Biogeochemistry, Brazilian Agricultural Research CorporationManaus, Brazil

**Keywords:** methane oxidation, Amazonian Dark Earth, terra preta de índio, methanotroph, *pmoA*, USC-α, *Methylocystis*

## Abstract

The oxidation of atmospheric CH_4_ in upland soils is mostly mediated by uncultivated groups of microorganisms that have been identified solely by molecular markers, such as the sequence of the *pmoA* gene encoding the β-subunit of the particulate methane monooxygenase enzyme. The objective of this work was to compare the activity and diversity of methanotrophs in Amazonian Dark Earth soil (ADE, Hortic Anthrosol) and their adjacent non-anthropic soil. Secondly, the effect of land use in the form of manioc cultivation was examined by comparing secondary forest and plantation soils. CH_4_ oxidation potentials were measured and the structure of the methanotroph communities assessed by quantitative PCR (qPCR) and amplicon pyrosequencing of *pmoA* genes. The oxidation potentials at low CH_4_ concentrations (10 ppm of volume) were relatively high in all the secondary forest sites of both ADE and adjacent soils. CH_4_ oxidation by the ADE soil only recently converted to a manioc plantation was also relatively high. In contrast, both the adjacent soils used for manioc cultivation and the ADE soil with a long history of agriculture displayed lower CH_4_ uptake rates. Amplicon pyrosequencing of *pmoA* genes indicated that USCα, *Methylocystis* and the tropical upland soil cluster (TUSC) were the dominant groups depending on the site. By qPCR analysis it was found that USCα *pmoA* genes, which are believed to belong to atmospheric CH_4_ oxidizers, were more abundant in ADE than adjacent soil. USCα *pmoA* genes were abundant in both forested and cultivated ADE soil, but were below the qPCR detection limit in manioc plantations of adjacent soil. The results indicate that ADE soils can harbor high abundances of atmospheric CH_4_ oxidizers and are potential CH_4_ sinks, but as in other upland soils this activity can be inhibited by the conversion of forest to agricultural plantations.

## INTRODUCTION

Most soils in the Amazon region have low fertility. Typically, Amazonian soils are acidic, have low P contents, low cation exchange capacity and high levels of Al at levels that can be toxic to crops (Cochrane and Sanchez, 1982). In contrast, Amazonian Dark Earth (ADE) soils, also known as terra preta de índio, are fertile soil patches found dispersed throughout the Amazon that were formed by the ancient Amazonian indigenous populations. It is believed that these soils were unintentionally or intentionally formed by long-term habitation with casual addition of domestic refuse and by long-lasting agricultural activity based on the clearing of vegetation and the incomplete combustion of organic material (Smith, 1980; Denevan, 1998; Glaser, 1999). Unlike their adjacent soils, ADE have high contents of P, Ca, Mg, Zn, Mn, and stable organic matter (Costa and Kern, 1999; Woods and McCann, 1999).

Differences in bacterial community structure and composition have been observed under different land use systems in Amazonian soils (Jesus et al., 2009; Navarrete et al., 2010; Taketani et al., 2013), which will in turn influence ecosystem processes such as the decomposition of organic matter and nutrient mineralization or its immobilization (Neher, 1999). In addition, the bacterial communities in ADE soils were shown by 16S rRNA tag sequence analysis to be distinct from their adjacent soils, particularly when compared at taxonomic levels lower than phylum (Taketani et al., 2013). One of the possible influences on the microbial communities of ADE soils is the presence of large amounts of biochar, which have prompted research into the effect of biochar application on microbial community structure and composition (Anderson et al., 2011; Khodadad et al., 2011). Replicating the high carbon and biochar contents of ADE in other soils has been suggested as a mechanism of CO_2_ sequestration (Sombroek et al., 2003; Lehmann, 2007); however, the presence of these relatively large amounts of carbon in ADE then raises concern whether changes in climate and land use may result in increased emissions of CO_2_ and CH_4_. One possible mechanism of increased CH_4_ emissions would be the decomposition of labile components of biochar to form substrates for methanogens (Knoblauch et al., 2008). To our knowledge, CH_4_ cycling in ADE soil has not been investigated and one important question is whether methane-oxidizing bacteria (methanotrophs) are present and active in ADE soils. If present, methanotrophs could consume atmospheric CH_4_ or potentially mitigate the release to the atmosphere of CH_4_ produced endogenously in the soil.

Upland soils, defined as those that are typically well-drained and oxic, have an important role in the global CH_4_ cycle by acting as a sink for atmospheric CH_4_ (King, 1992), which globally is estimated at more than 30 Tg y^-1^ (Denman et al., 2007). Although this activity is found in a wide variety of upland soils, pristine forest soils have been identified as the most efficient biological sinks of atmospheric CH_4_ (Dunfield, 2007; Dalal and Allen, 2008). Numerous studies have shown that the conversion of pristine land to agriculture lowers the oxidation capacity of the soil (Keller et al., 1990; Mosier et al., 1991; Hütsch et al., 1994; Jensen and Olsen, 1998; Priemé and Christensen, 1999; Knief et al., 2005; Levine et al., 2011). Various factors associated with agriculture have been shown to inhibit atmospheric CH_4_ oxidation, including soil compaction, acidification and fertilization (Dunfield, 2007). Conversely, the abandonment of agriculture can also lead to at least partial recovery of methanotroph populations and atmospheric CH_4_ uptake (Levine et al., 2011). ADE soils are commonly found on well-drained areas of the Amazon region (*terra firme*), and may also be sinks for atmospheric CH_4_.

Methanotroph diversity and activity has been assessed in different upland soils exhibiting atmospheric CH_4_ oxidation (Dunfield, 2007; Kolb, 2009). The diversity of atmospheric CH_4_ oxidizers is typically assessed by the detection of the *pmoA* gene, which encodes the β-subunit of methane monooxygenase (pMMO) enzyme (McDonald et al., 2008; Semrau et al., 2010). For the most part, as yet uncultivated microorganisms mediate atmospheric CH_4_ oxidation and are characterized by their *pmoA* gene sequences alone. In addition, phospholipid fatty acids have been used to identify atmospheric CH_4_ oxidizers (Bodelier et al., 2009). The USCα *pmoA* clade is widely distributed in upland soils (Knief et al., 2003) and based on gene analyses are believed to belong to Alphaproteobacteria most closely related to *Methylocapsa* (Ricke et al., 2005). The USCγ *pmoA* clade is another associated with upland soils exhibiting atmospheric CH_4_ uptake, and appear to favor neutral or somewhat alkaline soils (Knief et al., 2003). Another clade termed JR3, initially identified in grassland soil (Horz et al., 2005) was found to dominate in desert soils with atmospheric CH_4_ oxidation capacity (Angel and Conrad, 2009). *Methylocystis*-related species have been shown to use CH_4_ at relatively low concentrations (Knief and Dunfield, 2005; Knief et al., 2006; Baani and Liesack, 2008), but whether they are important consumers of atmospheric CH_4_ in upland soils is not clear.

To our knowledge, no studies have previously examined CH_4_ oxidation or the diversity of methanotrophs in ADE soils. The primary objective of this study was to determine the extent to which ADE soils are a potential sink for atmospheric CH_4_ and secondly to determine how the methanotroph community structure and their CH_4_ uptake potential compares between forested and agricultural sites.

## MATERIALS AND METHODS

### STUDY AREA, SOIL SAMPLING, AND SOIL ANALYSIS

Soil samples were collected from two different areas, Caldeirão and Barro Branco. The Caldeirão experimental research station from Embrapa Amazôonia Ocidental is located in Iranduba County in the Brazilian Central Amazon (03°26′ 00^′′^ S, 60°23′ 00^′′^ W). The other sampling area near the Barro Branco community is located in the Manacapuru County in the Brazilian Central Amazon (03°18′ 12^′′^ S, 60°31′ 45^′′^ W). ADE soils and their adjacent soils were collected from both areas. In both cases, the distance between the ADE soil zone and the adjacent soil zone was ∼2 km.

The soils were classified based on the World Reference Base for Soil Resources (FAO, 1998). ADE soils were classified as Hortic Anthrosol (i.e., reference horizon that results from prolonged habitation with casual additions of domestic organic refuse and cultural material). The adjacent soil from Caldeirão was classified as Haplic Acrisol (i.e., clay-rich soils with low fertility and toxic amounts of Al). The adjacent soil from Barro Branco was classified as Oxisol (i.e., red or yellowish soils with <10% weatherable minerals and low cation exchange capacity). At both areas, ADE soil and adjacent soil were sampled from secondary forest sites and agricultural sites cultivated with manioc (*Manihot esculenta*). The forested ADE and adjacent soil sites at Caldeirão were under ∼40-year-old secondary forest stands. At Barro Branco, the secondary forests were about 20 years-old. The agricultural sites in ADE and adjacent soils at Caldeirão had been used for manioc cultivation for at least 40 years, whereas the sites at Barro Branco had been deforested 5-years previously for conversion to plantations.

Soil samples were collected in February 2013. Three environmental replicates were collected from each sampling site. The sample plot (location) at each site was determined by choosing a random point, and from this reference point three sampling points (sublocations) 5 m apart were chosen for the collection of intact soil cores of 5 cm in diameter and 15 cm in length. Soil samples were collected in triplicate from each sublocation, which were subsequently homogenized to produce a composite soil sample for each sublocation. A total of 24 samples corresponding to the four sites (forested ADE, cultivated ADE, forested adjacent, and cultivated adjacent) from each of the two areas (Caldeirão and Barro Branco) were prepared. The samples for DNA extraction were transported from the field to the laboratory in an insulated box with dry ice. Approximately 1 kg of soil samples were collected from each of the 24 sublocations and sent to the department of Soil and Plant Nutrition of Embrapa Western Amazon. The frozen and unsieved soil samples were used for DNA extraction, whereas the 1 kg samples of fresh soil were sieved (2 mm mesh diameter) and used for the determination of soil chemical properties and CH_4_ oxidation potentials. Soil pH (H_2_O, 1:1), soil extractable Al, Ca, Fe, K, Mg, Mn, P, Zn, soil organic carbon (SOC), total C, total N, and cation exchange capacity were determined according to the methods described by Embrapa (1997).

### CH_4_ OXIDATION

Potential CH_4_ oxidation rates were measured using soil from each sampling point (sublocation). Ten grams of fresh sieved soil was placed into a 120 ml serum vial in duplicate (Bull et al., 2000; Horz et al., 2002; Shrestha et al., 2012). The bottles were sealed with butyl rubber stoppers, and final mixing ratios of 10, 100, 1000, and 10 000 ppmv of CH_4_ was injected into the gas headspace of the vials. The incubation of soil microcosms was performed at 25°C in the dark with shaking at 150 rpm for up to 19 days. CH_4_ concentrations were measured on a daily basis by gas chromatography with a flame ionization detector using 0.5 ml gas samples from the bottle headspaces, as described previously (Shrestha et al., 2012). CH_4_ oxidation rates were calculated by linear regression of CH_4_ consumption versus time for the incubations with 10 ppm CH_4_.

### DNA EXTRACTION FROM SOIL SAMPLES

Soil DNA extractions were carried out in triplicate from 0.3 *g* wet weight subsamples of each soil sample. Extractions were performed using the Nucleospin soil DNA extraction kit (Macherey-Nagel, Düren, Germany) according to the manufacturer’s instructions. DNA was quantified using a Qubit dsDNA HS Assay (Molecular Probes, Invitrogen, USA). The triplicate DNA extracts of each sampling sublocation were pooled.

### REAL-TIME QUANTITATIVE PCR ASSAYS

Real-time quantitative PCR (qPCR) with three technical replicates for each sublocation DNA sample was performed to determine the copy numbers of the *pmoA* genes. The qPCR assay using the primer set A189f-mb661r was used to target the conventional *pmoA* genes of *Methylocystaceae* and *Methylococcaceae* methanotrophs (Costello and Lidstrom, 1999; Kolb et al., 2003). The assay using primers A189f-Forest675r was used to target USCα *pmoA* genes (Kolb et al., 2003). The qPCRs were performed with the SYBR Green JumpStart Taq ReadyMix System (Sigma, Taufkirchen, Germany) on an iCycler instrument (Bio-Rad, Munich, Germany). The data were analyzed using Bio-Rad CFX Manager (version 3.0) software. PCR mixtures and thermal cycling conditions were performed as described previously by Kolb et al. (2003). Briefly, the A189f-Forest675r assay was performed in 25 μl reaction mixtures containing 12.5 μl of SYBR Green Jump-Start Taq Ready Mix (Sigma), 1 μM of each primer, 50 ng of BSA (Roche, Mannheim, Germany), and 4 mM MgCl_2_ (Sigma). The assay for the abundance of conventional *pmoA* genes (A189f-mb661r) was performed in 25-μl reaction mixtures containing 12.5 μl of SYBR Green Jump-Start Taq Ready Mix (Sigma), 0.667 μM of each primer and 4 mM MgCl_2_. Standards for qPCR were generated by serial dilution of stocks of a known number of plasmids containing a single cloned copy of a *Methylococcus pmoA* gene or a USCα *pmoA* gene, according to the assay. All samples from an experiment were run on a single plate.

### HIGH-THROUGHPUT SEQUENCING AND ANALYSIS

PCR was performed using the primers A189f and A682r that amplify a broad range of *pmoA*, *amoA,* and related sequences (Holmes et al., 1995; Lüke and Frenzel, 2011). The PCR components and conditions were identical to that described previously (Angel and Conrad, 2009). Briefly, the 50 μl reaction contained 5 μl of 10x AccuPrime^TM^ PCR Buffer II (Invitrogen, Karlsruhe, Germany), additional 1.5 mM MgCl_2_ (to a final concentration of 3 mM), 0.5 mM of each primer (Sigma), 50 ng of BSA (Roche) and 1 μl of Taq DNA polymerase (Invitrogen). All ADE samples could be amplified directly with the barcoded primer sets; however, it was not possible to obtain amplicons of the expected size for the adjacent soil samples using these primers. Therefore, a 2-step PCR procedure in which conventional primers (i.e., without barcodes) was used in the first step followed by a successive low-cycle-number amplification using the barcoded primers, as described by Berry et al. (2011). This approach successfully produced PCR amplicons of the expected size. To allow comparisons, the same 2-step PCR approach was used for all samples. Five replicate PCR reactions were performed for each sample. After amplification, PCR reactions were pooled and loaded on 1% agarose gel stained in GelRed^TM^ (Biotium Inc., Hayward, CA, USA). The DNA fragment of the correct size was excised from the agarose gel and eluted in 30 μl H_2_O using the QIAquick gel extraction kit (Qiagen, Hilden, Germany). The purified PCR products from all samples were mixed in a 1:1 ratio and sequenced at the Max Planck-Genome-Centre Cologne (Cologne, Germany) using a Roche 454 Genome Sequencer FLX System.

A detailed description of the procedures used for sequence analysis was described previously (Dumont et al., 2014). In this study, only sequences with read lengths longer than 300 bp were used for further analysis. The sorting of sequences according to barcodes, trimming and quality filtering were processed using mothur version 1.29.2 (Schloss et al., 2009). Chimeric sequences were identified and removed using uchime (Edgar et al., 2011) implemented in mothur. Classification of *pmoA* sequences was performed using standalone TBLASTN version 2.2.26+ against a curated database of *pmoA* sequences and the lowest common ancestor (LCA) algorithm in MEGAN version 4.70.4 (Huson et al., 2011), as described previously (Dumont et al., 2014). A total of 110,437 sequences were obtained. 42,213 reads (a range from 9022 to 2977 reads per library) remained after basic quality filtering. The amplification of non-target sequences is common with these primers (Bourne et al., 2001) and these contaminants were identified by an absence of similarity to the reference database and removed from further analysis. The contaminants corresponded to an average of 57% from ADE samples and 87% from adjacent soil samples. A total of 13,595 reads remained after removing these contaminant sequences, corresponding to an average of 2802 reads from ADE and 597 from adjacent soil samples.

Representative sequences from each *pmoA* clade identified during the sequence analysis were selected for further analysis. These reads were translated into amino acid sequences and added to a reference *pmoA/amoA* phylogenetic tree using parsimony in ARB (Ludwig et al., 2004).

Sequences are available through the Metagenomics Rapid Annotation (MG-RAST) server ^1^ with accession numbers 4577576.3 (TPISFBB2), 4577577.3 (TPISFBB3), 4577578.3 (TPISFBB4), 4577570.3 (TPIMBB2), 4577571.3 (TPIMBB3), 4577572.3 (TPIMBB5), 4577565.3 (ADJSFBB2), 4577566.3 (ADJSFBB3), 4577560.3 (ADJMBB2), 4577561.3 (ADJMBB3), 4577562.3 (ADJMBB4), 4577579.3 (TPISFC3), 4577580.3 (TPISFC4), 4577581.3 (TPISFC5), 4577573.3 (TPIMC2), 4577574.3 (TPIMC3), 4577575.3 (TPIMC4), 4577567.3 (ADJSFC2), 4577568.3 (ADJSFC4), 4577569.3 (ADJSFC5), 4577563.3 (ADJMC3), 4577564.3 (ADJMC4).

### STATISTICS

Differences in soil chemical properties were tested by one-way analysis of variance. Two-way analysis of variance model was used to assess differences in *pmoA* gene abundances between land uses and soil types. Test of proportions was used to observe significance of proportion difference in *pmoA* gene relative abundance generated by amplicon pyrosequencing between ADE and adjacent soils using prop.test in the R Stats Package ^2^. Significance level of *p* < 0.05 was applied for all statistical analyses and performed using R version 3.03 (R Foundation for Statistical Computing).

## RESULTS

### SOIL CHEMICAL PROPERTIES

The soil chemical properties are presented in **Table 1**. As previously reported, the measured soil chemical properties at the Caldeirão Experimental Station showed a clear distinction between ADE and adjacent soil samples (Taketani et al., 2013; Brossi et al., 2014). ADE soils from Barro Branco had similar properties to those at Caldeirão, with relatively high pH, Ca, CEC, K, Mg, Mn, P, SOC, and Zn compared to their adjacent soils. These characteristics indicate the potential for high agricultural productivity. In contrast, the adjacent soils (i.e., Haplic Acrisol and Oxisol) had lower pH and higher Al and Fe.

**Table 1:** Soil chemical properties of Amazonian Dark Earth (ADE) and their adjacent (ADJ) soils under secondary forest and manioc cultivation.

Soil properties	Amazonian Dark Earth				Adjacent soil				Statistics
	Secondary forest		Manioc plantation		Secondary forest		Manioc plantation		ADE vs. ADJ
	Barro Branco	Caldeirão	Barro Branco	Caldeirão	Barro Branco	Caldeirão	Barro Branco	Caldeirão	
Al^a^	0.03 ± 0.03^b^	0.01 ± 0	0.09 ± 0.08	0.16 ± 0.13	2.01 ± 0.26	1.80 ± 0.08	2.51 ± 0.35	1.37 ± 0.08	***^c^
Ca	5.19 ± 1.35	2.79 ± 0.50	3.45 ± 0.41	3.36 ± 0.11	0.17 ± 0.06	0.09 ± 0.04	0.23 ± 0.17	0.32 ± 0.06	***
CEC^†^	6.64 ± 1.24	3.49 ± 0.51	4.50 ± 0.47	5.64 ± 0.29	2.32 ± 0.23	2.06 ± 0.11	2.90 ± 0.17	1.89 ± 0.03	***
Fe	5.00 ± 1.00	26.33 ± 1.53	13.00 ± 3.46	51.33 ± 3.51	112.33 ± 19.30	313.00 ± 53.26	75.33 ± 31.21	259.67 ± 26.84	**
K	21.00 ± 1.00	44.67 ± 5.03	20.33 ± 3.79	20.33 ± 6.50	15.33 ± 1.15	19.67 ± 5.69	16.33 ± 0.58	17.67 ± 3.51	*
Mg	1.36 ± 0.09	1.33 ± 0.26	0.90 ± 0.16	1.08 ± 0.31	0.09 ± 0.01	0.08 ± 0.03	0.09 ± 0.03	0.15 ± 0.01	***
Mn	48.65 ± 9.40	52.1 ± 12.04	31.63 ± 3.24	22.02 ± 2.85	3.9 ± 0.04	1.88 ± 0.75	4.93 ± 0.33	1.84 ± 0.29	**
P	26.00 ± 9.54	56.33 ± 12.66	51.33 ± 13.86	73.33 ± 4.50	6.00 ± 0.64	5.67 ± 1.53	5.33 ± 2.08	1.33 ± 0.58	***
pH_water_	5.63 ± 0.13	5.85 ± 0.27	5.53 ± 0.13	5.30 ± 0.29	4.33 ± 0.06	3.84 ± 0.09	4.38 ± 0.08	4.33 ± 0.13	**
SOC	29.90 ± 1.39	32.31 ± 3.36	24.7 ± 2.50	13.15 ± 1.34	19.23 ± 0.97	11.72 ± 1.88	21.2 ± 1.11	10.84 ± 0.25	**
Total C	3.23 ± 0.45	2.99 ± 0.32	3.07 ± 0.33	2.14 ± 0.05	2.71 ± 0.43	2.12 ± 0.36	2.33 ± 0.55	1.81 ± 0.26	*
Total N	0.24 ± 0.08	0.25 ± 0.04	0.24 ± 0.02	0.16 ± 0.01	0.19 ± 0.02	0.17 ± 0.02	0.16 ± 0.02	0.14 ± 0.02	ns
Zn	10.74 ± 1.96	6.76 ± 0.49	4.40 ± 0.69	2.32 ± 0.28	5.63 ± 1.15	0.32 ± 0.03	5.63 ± 1.15	0.51 ± 0.16	**

*^†^Cation exchange capacity.*

*^a^Al, Ca, CEC, and Mg are expressed in centimoles per cubic decimeter; Fe, K, Mn, P, and Zn are expressed in milligram per cubic decimeter; soil organic C (SOC) is expressed in gram per kilogram; Total C and Total N in percentage.*

*^b^Values are means (*n* = 3) followed by the standard deviation.*

^c^ANOVA, *n* = 12, *p < 0.05, **p < 0.01, ***p < 0.001, ns indicates *p* ≥ 0.05.

### SOIL CH_4_ OXIDATION POTENTIALS

CH_4_ oxidation was immediate at concentrations of 10 and 100 ppmv, but a lag phase of 6–10 days was observed for concentrations of 1000 and 10,000 ppmv (results not shown). Relatively high rates of high-affinity CH_4_ oxidation (10 ppm CH_4_) were observed in all soils from the forested sites and the ADE soil used for manioc cultivation at the Barro Branco area (**Table 2**). In contrast, the CH_4_ oxidation rates were more than one-order of magnitude lower in both plantations in adjacent soil and the ADE plantation soil at Caldeirão. The precise history of these soils is not available, but members of the local communities indicated that manioc has been cultivated in ADE soil at the Caldeirão site for living memory (>40 years), whereas the Barro Branco ADE soil was only recently (5 years) converted from forest to agriculture by slash-and-burn.

**Table 2:** CH_**4**_ oxidation rates in Amazonian Dark Earth and their adjacent soils under secondary forest and manioc cultivation.

Soil type and site	Land use	CH_4_ oxidation rate^a^ [pmol of CH_4_ (*g* dw)^--1^ h^--1^]
Amazonian Dark Earth
Barro Branco	Secondary forest	33.5 ± 1.6
Caldeirão	Secondary forest	48.1 ± 4.5
Barro Branco	Manioc plantation	50.0 ± 1.4
Caldeirão	Manioc plantation	6.0 ± 1.1
Adjacent soil
Barro Branco	Secondary forest	31.0 ± 1.9
Caldeirão	Secondary forest	21.0 ± 1.7
Barro Branco	Manioc plantation	8.0 ± 1.9
Caldeirão	Manioc plantation	6.1 ± 2.2

*^a^Errors are standard deviation (*n* = 3).*

### ABUNDANCE OF METHANOTROPHS

**FIGURE 1:**
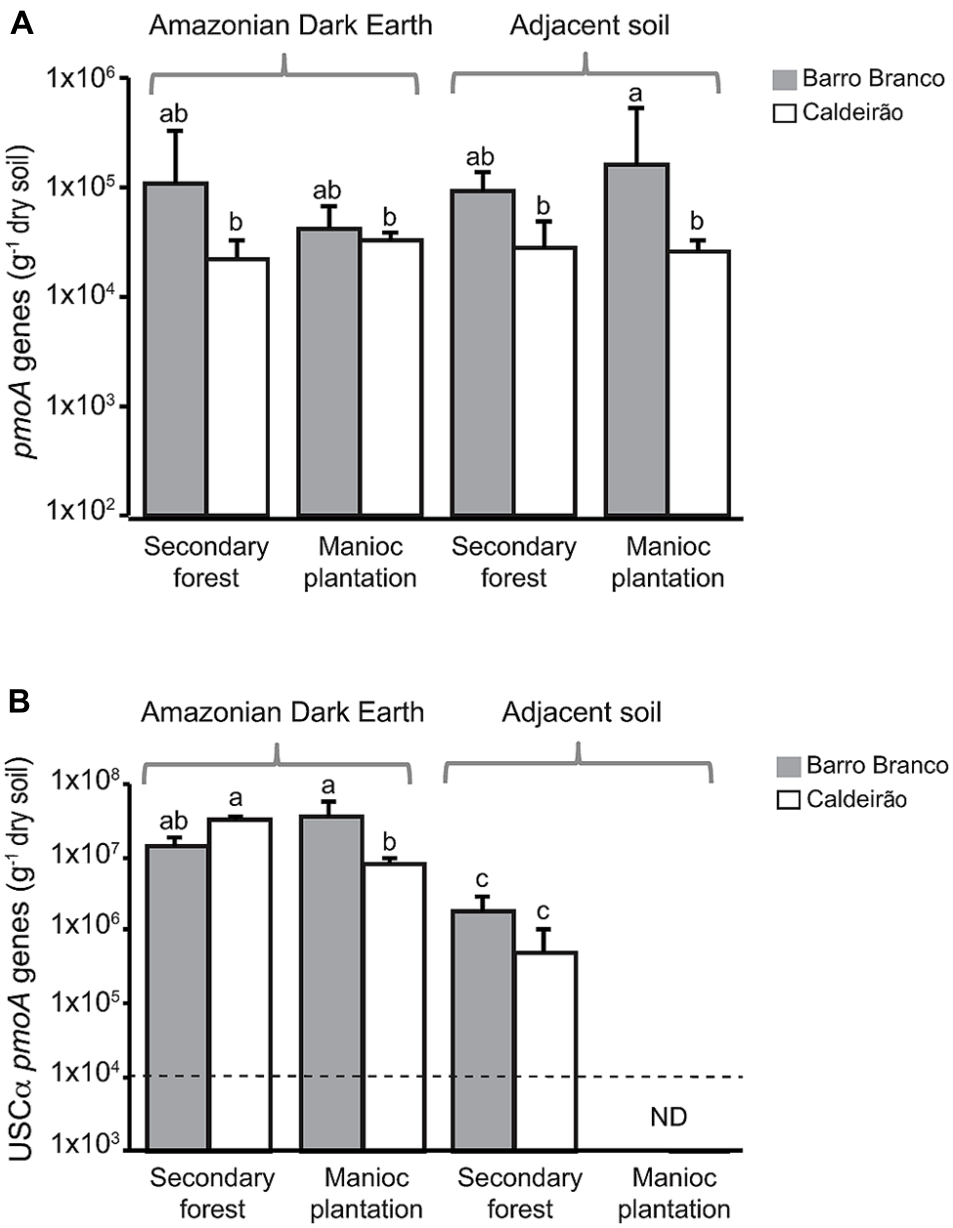
**Methanotroph *pmoA* abundances in Amazonian Dark Earth and adjacent soils under secondary forest and manioc cultivation at Barro Branco and Caldeirão sites. (A)** Abundance of *pmoA* genes using the A189f-mb661r qPCR assay targeting conventional *pmoA* genes (i.e., mostly *Methylocystis* in these soils). **(B)** Abundance of USCα *pmoA* genes determined with the A189f-Forest675r qPCR assay. Abundances denoted with different letters above the bars are significantly different from each other (Tukey’s HSD, *p* < 0.05). ND indicates that the target gene was below the detection limit of the qPCR assay, which is indicated by the dashed line.

**FIGURE 2:**
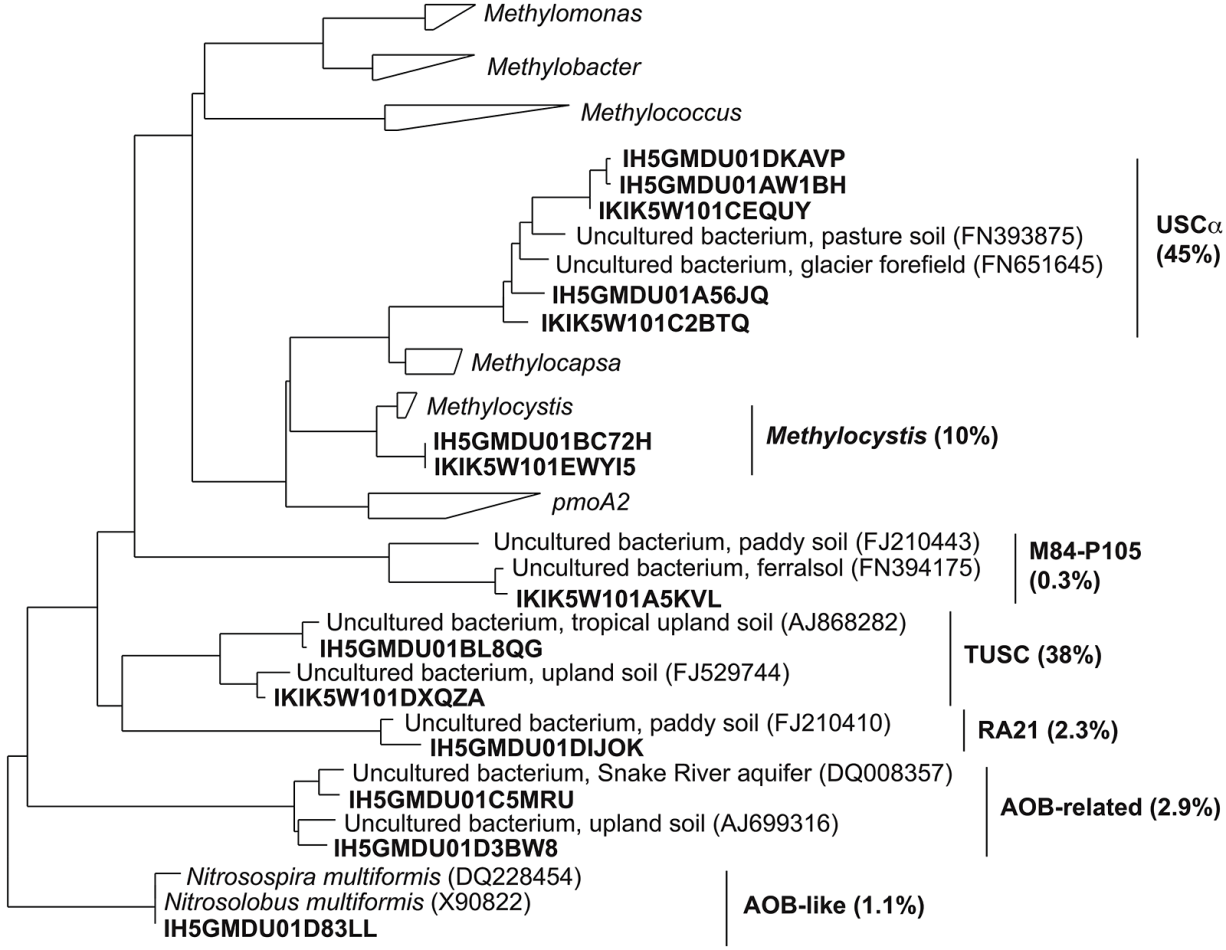
**Phylogenetic tree of representative *pmoA* pyrosequences (bold type) obtained in this study.** The percentages of sequences corresponding to each clade are shown in parentheses.

Quantitative real-time PCR assays were used to determine the copy numbers of *pmoA* genes in ADE and adjacent soils from both secondary forest and the manioc cultivation sites (**Figure 1**). The *pmoA* qPCR assay with primers A189f-mb661r targets methanotrophs belonging to the *Methylococcaceae* and *Methylocystaceae* families and generally has poor specificity for the genes from other families of methanotrophs. The abundance of genes detected with this assay (**Figure 1A**) was not significantly affected by soil type or land use. Based on the diversity of *pmoA* genes detected in the soils (**Figure 2**), these results correspond to *Methylocystis pmoA* genes. Another qPCR assay was used to specifically enumerate USCα *pmoA*, which are a common uncultivated group associated with atmospheric CH_4_ oxidation. In ADE soils, the abundances of USCα *pmoA* (**Figure 1B**) were more than two-orders of magnitude higher than *Methylocystis pmoA* genes (**Figure 1A**). USCα were below the detection limit (1 × 10^4^ copies *g*^-1^ dry weight soil) in the plantations of adjacent soils. Taking the data from Barro Branco and Caldeirão sites together, the abundance of USCα *pmoA* was significantly higher in ADE than adjacent soil (ANOVA, *p* < 0.0001), but the difference in abundance based on land use (forested versus cultivated) was not significant (ANOVA, *p* = 0.77).

### COMPOSITION OF METHANOTROPH COMMUNITIES

The methanotroph communities in the soils were analyzed by *pmoA* gene pyrosequencing. PCR using the A189f-A682r primer combination retrieves diverse *pmoA*-related genes, including the proteobacterial *pmoA* genes and those from uncultivated methanotrophs believed to be responsible for atmospheric CH_4_ uptake in upland soils (McDonald et al., 2008). A known problem with these primers is a tendency to co-amplify non-specific sequences, which can make clone libraries useless (Bourne et al., 2001). Non-specific amplification with these primers was also observed in our pyrosequencing data, with an average of 87% of reads from adjacent soils corresponding to non-target reads. The advantage of relatively high number of reads obtainable by pyrosequencing compared with clone libraries meant that sufficient numbers of genuine *pmoA* sequences were still available to allow for comparisons in *pmoA* diversity between the samples.

Almost all sequences passing the quality-filtering steps were assigned to seven clades, which were defined and described previously (Lüke and Frenzel, 2011). Representative sequences from each of these clades were added to a database of *pmoA* and *amoA* sequences and are shown in a simplified phylogenetic tree (**Figure 2**). The most abundant clades identified were USCα, tropical upland soil cluster (TUSC) and *Methylocystis*. The other less abundant clades were RA21, M84-P105, AOB-rel, and the AOB-like group. AOB-rel is also referred to in the literature as Cluster 1 (Kolb et al., 2005).

**FIGURE 3:**
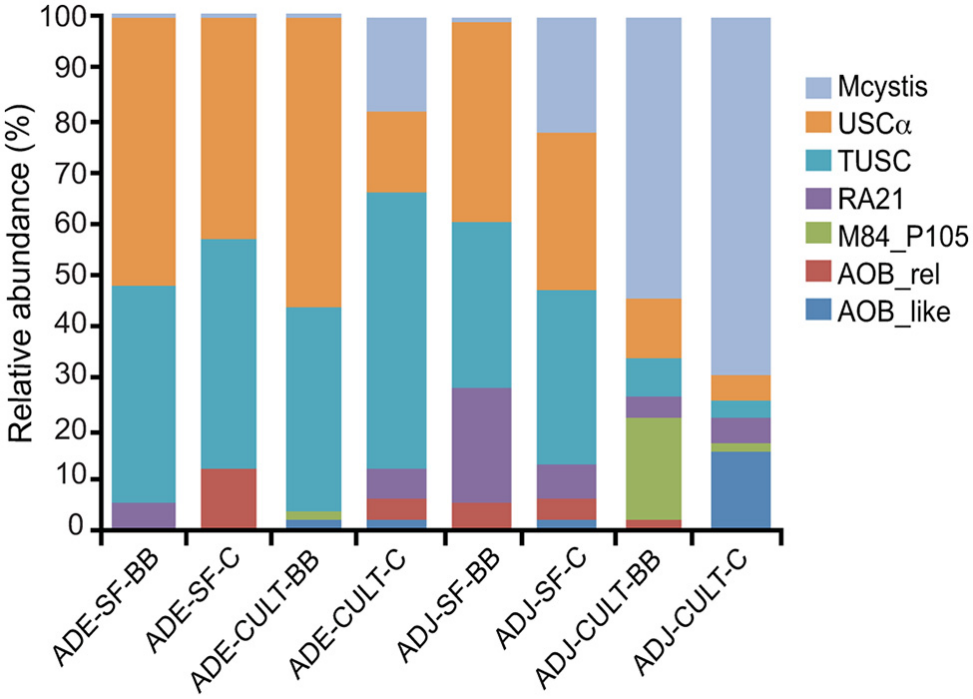
**Relative abundance of detected *pmoA*-related clades obtained by amplicon pyrosequencing of Amazonian Dark Earth (ADE) and adjacent soils (ADJ) under secondary forest (SF) and manioc cultivation (CULT) at Barro Branco (BB) and Caldeirão (C) sites.** The sequence clades are described in the text. “*Mcystis*” indicates* Methylocystis*.

The relative abundance of the clades from each of the sites is shown in **Figure 3**. A test of proportions indicated that, with the exception of AOB-like sequences, the relative abundances of these clades were significantly different (*p* < 0.05) between the ADE and the adjacent soils (Table S1).

### COMPARISON OF RELATIVE *pmoA* GENE ABUNDANCES OBTAINED BY qPCR AND PYROSEQUENCING

**FIGURE 4:**
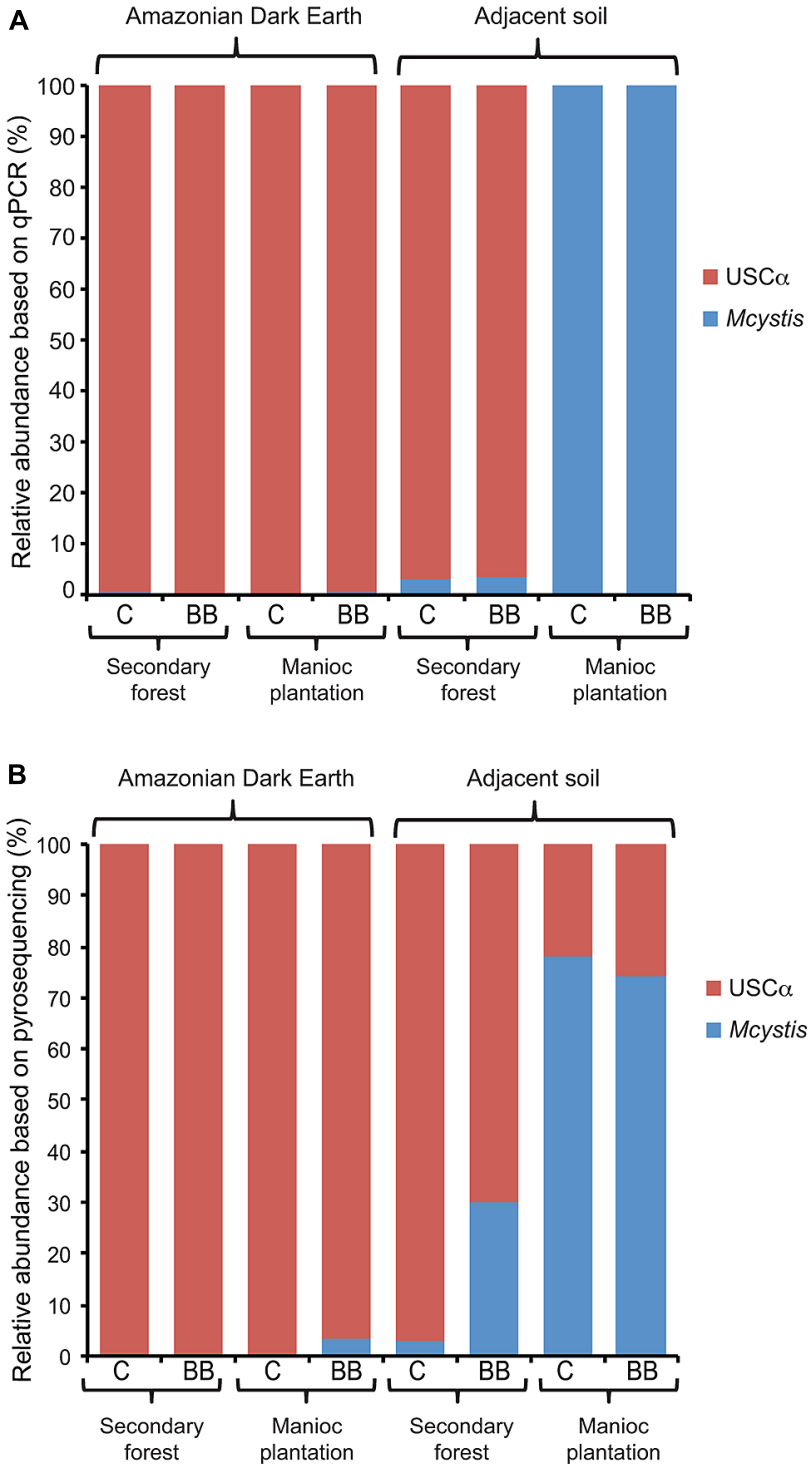
**Relative abundance of *Methylocystis* (*Mcystis*) and USCα *pmoA* genes in Amazonian Dark Earth and their adjacent soils under secondary forest and manioc cultivation at Barro Branco (BB) and Caldeirão (C) sites.** Abundances calculated based on **(A)** qPCR or **(B)** pyrosequencing data.

Data from the *pmoA* qPCR assays and amplicon pyrosequencing approaches provided independent numbers to compare the relative abundance of *pmoA* clades in the soils. Based on the diversity of *pmoA* detected by pyrosequencing, *Methylocystis* was the only group present that was a target for the A189f-mb661r *pmoA* qPCR assay. Therefore, the abundance of *pmoA* detected with this qPCR assay was taken as the abundance of *Methylocystis pmoA* genes. Calculating the relative abundance of *Methylocystis* and USCα from the qPCR assays (**Figure 4A**) and the pyrosequencing dataset (**Figure 4B**) showed relatively good agreement.

The major difference between these data was that USCα in the cultivated adjacent soils was below the detection limit of the qPCR assay (**Figure 1B**) and therefore its relative abundance was calculated as 0 (**Figure 4A**); however, USCα sequences were detected of ∼20% of *Methylocystis* in the pyrosequencing dataset from these samples (**Figure 4B**).

## DISCUSSION

Many processes, such as CH_4_ oxidation, are crucial for soil ecosystem functioning and have an impact on global biogeochemistry. Forest soils in particular have been identified as an efficient sink for atmospheric CH_4_ and are highly sensitive to land use change (Dunfield, 2007). Here, we have characterized methanotrophs in ADE and their adjacent soils (Haplic Acrisol and Oxisol) under two different land uses (i.e., secondary forest and manioc cultivation). These approaches showed two major outcomes with respect to ADE soils: (1) high CH_4_ oxidation rates were observed in three of four ADE soils examined, and (2) high relative and absolute abundances of methanotrophs belonging to the USCα *pmoA* cluster associated with atmospheric CH_4_ oxidation in upland soils were observed in all ADE soil samples, independent of land use.

### CH_4_ OXIDATION POTENTIALS

The CH_4_ oxidation rates were relatively high in forested sites. This is in agreement with other studies of tropical forests soils (Verchot et al., 2000; Veldkamp et al., 2008; Zhang et al., 2008; Dörr et al., 2010). Surprisingly, the ADE soil at the Barro Branco site under manioc cultivation showed a CH_4_ oxidation rate similar to that of the forested sites. Many studies have shown that conversion of forest to agriculture diminishes CH_4_ uptake. For example, after 2 years of agriculture a Norwegian soil showed a fivefold decrease in CH_4_ oxidation rate (Jensen and Olsen, 1998). At the time of sampling, the ADE soil at Barro Branco had been used for manioc cultivation for ∼5 years, suggesting that it too should have shown a decreased CH_4_ oxidation potential. The ADE soil at the manioc planation at the Caldeirão area, which has a longer history of cultivation, showed a decreased CH_4_ oxidation potential. The cultivated ADE site at Barro Branco had been burned to clear the land, which may have also influenced in CH_4_ oxidation capacity as in some cases fire has been shown stimulate atmospheric CH_4_ oxidation (Jaatinen et al., 2004).

### ABUNDANCE AND COMMUNITY COMPOSITION OF METHANOTROPHS

Differences in the methanotroph communities were found between ADE and adjacent soils under secondary forest and manioc cultivation, indicating that the methanotrophic community is altered depending on soil type and land use. USCα were the predominant methanotrophs in all ADE soils and the forested adjacent soils. This group is as yet uncultivated, but is believed to be responsible for atmospheric CH_4_ consumption in many forest soils (Dunfield, 2007; Kolb, 2009; Nazaries et al., 2013). The abundance of USCα* pmoA* genes was ∼1 × 10^7^ per gram dry weight in the ADE soils, which was one-order of magnitude higher than in the forested sites of the adjacent soils. In comparison, the same assay used to quantify USCα in a German forest soil detected ∼1 × 10^6^ gene copies per gram dry weight of soil (Kolb et al., 2005), suggesting that their abundance in ADE was relatively high.

It was surprising that USCα abundances were equally high in the cultivated and forested ADE soils (**Figure 1B**). This pattern was different for the adjacent soils where they were below the qPCR detection limit in the cultivated soils, indicating abundances at least two-orders of magnitude lower than the forested sites. In comparison, the manioc plantation in ADE soil at Caldeirão has a long history of agriculture use, yet the USCα abundance was only threefold lower than in the corresponding forested soil. To the best of our knowledge, this is the first study to detect a high absolute and relative abundance of USCα in agricultural soils. Priemé et al. (1997) showed that CH_4_ oxidation rates took more than 100 years to reach pre-cultivation levels and that the highest rates were in the oldest (200 years) woodlands. The apparent resilience of USCα populations in ADE soil compared with other upland soils, possibly from a protective property of ADE, suggests that recovery of CH_4_ oxidation capacity after agricultural abandonment might be faster in ADE than other types of upland soil.

Also of note in this study was that the CH_4_ uptakes rates were relatively low in cultivated ADE soil at Caldeirão, but USCα abundance in this soil was relatively high. One possible explanation for this lack of correlation is that USCα methanotrophs can incorporate acetate and possibly other organic carbon substrates (Pratscher et al., 2011), suggesting that CH_4_ oxidation is a facultative trait in these organisms and CH_4_ is oxidized only under certain conditions. Evidence that USCα are not obligate methanotrophs include reported failures to sufficiently label their nucleic acids with ^13^CH_4_ for stable isotope probing (Bengtson et al., 2009; Pratscher et al., 2011), and an ability of many of their closest cultivated relatives to grow using multicarbon compounds (Tamas et al., 2014). Another possibility is that the USCα methanotrophs in this ADE soil at Caldeirão have been able to remain dormant, or possibly that DNA from dead cells is relatively stable in ADE soil.

The diversity of methanotrophs observed in this study was similar to the observations of Dörr et al. (2010), who observed in Brazilian ferralsols a prevalence of USCα in natural and afforested sites and higher relative abundances of *Methylocystis* and *Methylococcus* spp. in agricultural soil under conventional farming. Among the cultivated methanotrophs, we only detected *Methylocystis pmoA* and no conventional *pmoA* genes from *Methylococcaceae* methanotrophs; however, the unconventional M84-P105 *pxmA* sequences, which have been shown to belong to members of the *Methylococceae* (Tavormina et al., 2011), were detected in cultivated adjacent soils suggesting a low abundance of these methanotrophs in some soils (**Figure 3**). Although the relative abundance of *Methylocystis* was high in the adjacent soils from manioc plantation sites (**Figure 3**), no difference in their absolute abundance between ADE and adjacent soil, or between forested and cultivated sites was observed at this sampling time (**Figure 1**). *Methylocystis* have been shown to be important consumers of CH_4_ in hydromorphic soils under dry conditions when CH_4_ concentrations are relatively low (Knief et al., 2005). These *Methylocystis* possess an unconventional pMMO gene, termed pMMO2 (Ricke et al., 2004), which is expressed under low CH_4_ (Baani and Liesack, 2008). We only detected two *pmoA2* gene sequences in our pyrosequencing dataset (data not shown), suggesting that conditions in these Amazonian soils at the time of this analysis were not favorable for pMMO2-possessing oligotrophic *Methylocystis* species.

Other *pmoA*-related gene sequences were detected, such as TUSC, AOB-rel and AOB-like groups. The AOB-like sequences correspond to the *amoA* genes of *Nitrosospira* and *Nitrosomonas* (**Figure 2**). In ADE soils, these *amoA* sequences were only detected in plantation soil, which is likely a consequence of enrichment by ammonium fertilizer applied to the soil for manioc cultivation. The TUSC and AOB-rel groups have not been linked to cultivated organisms and the function of the enzyme encoded by these genes is not known (Lüke and Frenzel, 2011). TUSC or “tropical upland soil cluster” is also termed “Cluster 2” elsewhere (Knief et al., 2005). As the name implies, they were found to be abundant in some tropical upland soils (Knief et al., 2005), but have also been detected in temperate forest soil (Knief et al., 2003). It is noteworthy that the relative abundance of TUSC tended to mirror USCα in these Amazonian soils. One possibility to explain this correlation is that TUSC sequences are a divergent *pmoA* gene found in USCα methanotrophs, such as the case with M84-P105 *pxmA* in *Methylomonas* and *pmoA2* in *Methylocystis*; however, other studies have not observed a correlation between USCα and TUSC relative abundances (Kolb, 2009; Dörr et al., 2010).

## CONCLUSION

This study has shown that ADE soils are a potential sink for atmospheric CH_4_. The relatively high rate of “high-affinity” CH_4_ uptake by the ADE soil with a 5-year history of agriculture contradicts many studies showing the process to be sensitive to land use change. All the ADE soils examined had a high abundance of USCα methanotrophs (∼10^7^ *pmoA* genes *g*^-1^ soil), which was particularly surprising for the ADE soil at the Caldeirão site that had a long history of manioc cultivation. In comparison, the abundance of USCα methanotrophs was up to 1000-fold lower in adjacent than ADE soil, and both the adjacent soils used for agriculture displayed relatively low CH_4_ uptake rates. This raises the question if USCα methanotrophs are indeed more resistant to disturbance in ADE than in other upland soils and whether this apparent resilience of ADE extends to the protection of other groups of vulnerable microorganisms and their associated functions.

## Conflict of Interest Statement

The authors declare that the research was conducted in the absence of any commercial or financial relationships that could be construed as a potential conflict of interest.

## Footnotes

1 http://metagenomics.anl.gov/

2 http://www.r-project.org
