# Supplementary material for: Activity and abundance of methane-oxidizing bacteria in secondary forest and manioc plantations of Amazonian Dark Earth and their adjacent soils
Source: Front Microbiol. 2014 Oct 22;5:550. doi: 10.3389/fmicb.2014.00550 (PMC4205850; doi:10.3389/fmicb.2014.00550)
Supplement: Supplementary file 1 [file Table_1.DOCX]

Table S1. Relative abundance of methanotroph populations obtained by *pmoA* amplicon pyrosequencing in Amazonian Dark Earth (ADE) and adjacent soils (ADJ) under secondary forest (SF) and manioc cultivation (CULT) at Barro Branco (BB) and Caldeirão (C) sites.

| *pmoA* gene | Amazonian Dark Earth | | | | |  | Adjacent soil | | | | |  | ADExADJ |
| --- | --- | --- | --- | --- | --- | --- | --- | --- | --- | --- | --- | --- | --- |
|  | Forest | |  | Cultivation | |  | Forest | |  | Cultivation | |  |  |
|  | BB | C |  | BB | C |  | BB | C |  | BB | C |  | p-value^b^ |
| AOB_like | 0.21^a^±0.36 | 0.04±0.07 |  | 2.87±4.77 | 2.42±3.67 |  | ND^c^ | 2.21±3.83 |  | 0.04±0.07 | 14.27±24.71 |  | 0.03 |
| AOB_rel | 0.86±1.39 | 11.86±10.70 |  | 0.08±0.07 | 4.64±7.51 |  | 5.29±7.93 | 4.43±7.67 |  | 2.34±3.95 | 0.53±0.92 |  | ns^d^ |
| Mcytis | 0.29±0.07 | 0.25±0.12 |  | 0.16±0.14 | 18.02±15.07 |  | 1.23±0.89 | 22.72±17.63 |  | 53.68±33.66 | 61.86±46.85 |  | 2.20e-16 |
| M84_P105 | ND | ND |  | 0.08±0.07 | ND |  | 0.41±0.38 | ND |  | 19.42±21.91 | 0.99±0.93 |  | 8.39e-06 |
| RA21 | 4.39±7.60 | 0.16±0.07 |  | 0.04±0.07 | 4.43±3.84 |  | 22.17±35.97 | 5.82±1.42 |  | 4.43±5.24 | 3.90±2.52 |  | 3.46e-05 |
| TUSC | 42.06±3.56 | 44.52±23.16 |  | 41.20±1.57 | 54.26±19.19 |  | 31.61±43.87 | 33.07±18.08 |  | 6.45±4.61 | 2.91±2.20 |  | 5.42e-15 |
| USCα | 52.20±3.49 | 43.09±20.13 |  | 55.56±4.86 | 16.02±8.15 |  | 39.13±43.80 | 29.61±5.03 |  | 13.43±8.41 | 4.55±0.77 |  | 1.09e-08 |

^a^Values are means (n=3) followed by the standard deviation

^b^Proportion test denotes statistical significance (p < 0.05) of proportion differences of *pmoA* gene relative abundance between ADE and ADJ

^c^Not detected

^d^Not significant
